# Epidemic characteristics, high-risk townships and space-time clusters of human brucellosis in Shanxi Province of China, 2005–2014

**DOI:** 10.1186/s12879-016-2086-x

**Published:** 2016-12-19

**Authors:** Qiulan Chen, Shengjie Lai, Wenwu Yin, Hang Zhou, Yu Li, Di Mu, Zhongjie Li, Hongjie Yu, Weizhong Yang

**Affiliations:** 1 Division of Infectious Disease, Key Laboratory of Surveillance and Early-warning on Infectious Disease, Chinese Center for Disease Control and Prevention, Beijing, People’s Republic of China; 2 Worldpop Project, Department of Geography and Environment, University of Southampton, Southampton, SO17 1BJ UK

**Keywords:** Human brucellosis, Epidemiology, Zoonosis, High-risk areas, Shanxi province, Space-time scan statistic, Poisson model, Geographic information system

## Abstract

**Background:**

Brucellosis, one of the world’s most important zoonosis, has been re-emerging in China. Shanxi Province, located in northern China, where husbandry development has been accelerated in recent years, has a rather high incidence of human brucellosis but drew little attention from the researchers. This study aimed to describe the changing epidemiology of human brucellosis in Shanxi Province from 2005 to 2014 and explore high-risk towns and space-time clusters for elucidating the necessity of decentralizing disease control resource to township level in epidemic regions, particularly in hotspot areas.

**Methods:**

We extracted data from the Chinese National Notifiable Infectious Disease Reporting System to describe the incidence and spatiotemporal distribution of human brucellosis in Shanxi Province. Geographic information system was used to identify townships at high risk for the disease. Space-Time Scan Statistic was applied to detect the space-time clusters of human brucellosis during the past decade.

**Results:**

From 2005 to 2014, a total of 50,002 cases of human brucellosis were recorded in Shanxi, with a male-to-female ratio of 3.9:1. The reported incidence rate increased dramatically from 7.0/100,000 in 2005 to 23.5/100,000 in 2014, with an average annual increase of 14.5%. There were still 33.8% cases delaying diagnosis in 2014. The proportion of the affected towns increased from 31.5% in 2005 to 82.5% in 2014. High-risk towns spread from the north to the center and then south of Shanxi Province, which were basins and adjacent highlands suitable for livestock cultivation. During the past decade, there were 55 space-time clusters of human brucellosis detected in high risk towns; the clusters could happen in any season. Some clusters’ location maintained stable over time.

**Conclusions:**

During the last decade, Shanxi province’s human brucellosis epidemic had been aggravated and high-risk areas concentrated in some towns located in basins and adjacent highlands. Space-time clusters existed and some located steadily over time. Quite a few cases still missed timely diagnosis. Greater resources should be allocated and decentralized to mitigate the momentum of rise and improve the accessibility of prompt diagnosis treatment in the high-risk townships.

**Electronic supplementary material:**

The online version of this article (doi:10.1186/s12879-016-2086-x) contains supplementary material, which is available to authorized users.

## Background

Brucellosis, a classic zoonosis that is endemic among more than 170 countries, causes not only heavy agriculturally economic losses but also great burden to human health [1]. Brucellosis is also one of the major neglected zoonotic diseases which needs engaging more control efforts, as containing neglected zoonotic diseases is an effective approach to poverty alleviation in livestock-keeping communities [2]. Human brucellosis is acquired mainly through contact with infected animals and consumption of contaminated animal products, especially raw goat or sheep milk and cheese [3, 4]. In some circumstances, human brucellosis may be acquired via inhalation of infectious aerosols, which is the main transmittal channel for laboratory-acquired infection [4, 5]. Occupational exposure is the main risk factor in the countries where the raw milk and its products are strictly pasteurized. The shepherds, breeders, abattoirs workers, veterinarians, diary-industry professionals and laboratory personnel dealing with this bacteria are regarded as the high risk group. *Brucella*, the cause of brucellosis, is also regarded as a biological weapon due to its capacity of aerosol transmission [6]. Human brucellosis can affect any organ or tissue and thus may present a variety of signs and symptoms such as fever, fatigue, nausea, vomiting, malaise, headache, myalgia, and arthralgia in the acute phase [7]. These signs and symptoms of brucellosis resembled other contagious diseases and thus easily lead to under diagnosis [1, 2]. Although human brucellosis is rarely fatal, delayed and inadequate treatment in acute phase may lead to chronic disease which is difficult to cure [1–4]. The case fatality rate of human brucellosis is less than 1%, with the majority of deaths attributed to endocarditis [4].

Cases of human brucellosis are confirmed by laboratory examination in combination with recent epidemiologic history and clinical features. Cases are diagnosed by isolation of the etiologic agent, *Brucella* spp., from bone marrow, blood, or other tissue. However, this culture technique is seldom used in endemic developing countries because it is hazardous and requires biosafety bench. Polymerase chain reaction methods are rapid and accurate to detect *Brucella* DNA, but still need further standardization for better quality control [3, 4]. Therefore, serologic tests are the primary method for diagnosis of brucellosis in most countries. The common serologic tests include the rose bengal plate test (RBPT), serum agglutination test (SAT), Coombs test, complement fixation test, and enzyme-linked immunosorbent assay (ELISA). Those serological tests are also applied to detect animal brucellosis. Generally, RBPT and SAT are used as screening tests and the rest as confirmation tests [7–11]. Brucellosis has a rather high proportion of relapse or initial treatment failure, mainly attributed to doctors’ inappropriate prescription and patients’ noncompliance rather than drug resistance [12, 13]. Immunization is the most effective approach to eliminate animal brucellosis. However, vaccination is seldom used to protect humans from the disease, due to safety concerns. Attenuated live vaccines have been widely used only in the former Soviet Union and China last century [1–4].

The incidence rate of human brucellosis varies across countries and even regions within the same country, ranging from 0.03/100,000 to 160/100,000 per year [4, 14]. However, underreporting of brucellosis is common worldwide because it is easily unrecognized by both patients and physicians due to its nonspecific clinical features [1–4]. The national incidence of human brucellosis in China has been dramatically increasing in recent years [15–23], reaching a historic high in 2014 with 57,222 reported cases and incidence rate of 4.2/100,000 (the population is 1367.8 million) [24]. A few high-risk counties were detected in the Inner Mongolia Autonomous Region and neighboring provinces of Shanxi, Heilongjiang, Jilin, and Xinjiang [15]. However, few studies have explored high-risk areas at the township level [15, 23–32]. Such investigation might reveal new specific characteristics of human brucellosis epidemic, so as to provide evidence for adjusting disease control strategies especially in endemic provinces. We therefore aimed to examine epidemic characteristics and detect high-risk areas of human brucellosis at the township scale in Shanxi, a province with a population of 36.5million, where the number of annual reported new case was 8540 in 2014, only second to Inner Mongolia Autonomous Area (with 10,135 annual reported new cases) among the 31 provinces in China [15, 18–20] but little attention was drawn from researchers [23–33].

## Methods

### Data source

All data related to human brucellosis were extracted from the Chinese National Notifiable Infectious Disease Reporting Information System (NIDRIS). As the authorized communicable disease surveillance system in China, the NIDRIS was initiated in the 1950s and has been continually improved, especially since the SARS outbreak in 2003. An internet-based NIDRIS were launched nationwide by Chinese government on January 1^st^ 2004 and it achieved timely online monitoring of individual disease cases, which was regarded as a milestone of the communicable diseases’ surveillance history in China [34].

Human brucellosis has been included as a notifiable infectious disease in China since 1955. All clinical practitioners are mandated to report any probable or confirmed cases of notifiable infectious disease to the local Center for Disease Control and Prevention (CDC). The CDC at the county level is the basic unit of case reporting and data management, and the township hospital and city community health center (CCHC) act as the subunit, jointly shouldering the routine activities of disease control and prevention. NIDRIS has covered all healthcare institutes including hospitals at all levels and CCHC in China. In 2005, 93.0% of hospitals at county or higher levels reported data to NIDRIS via the Internet, rising to 97.2% in 2014. Other hospitals reported data via a paper-based system to local CDCs by mail [35, 36]. During the study period, Notification of brucellosis cases fitting the relevant definition was mandatory. While the method of notification may impact on the timeliness of diagnosis, it is not felt that this impacts on ascertainment in this retrospective study.

#### Case definition

Brucellosis cases are classified as probable (clinically diagnosed) or confirmed (laboratory confirmed), based on the guidelines for diagnosis of human brucellosis issued by national health authority in 1995 and 2007, which were successively applied from 1996 to 2006 and from 2007 to 2014, respectively (Additional file 1). Probable cases of brucellosis are diagnosed with combination of epidemiologic exposure, clinical manifestations, and/or positive results of presumptive laboratory tests including RBPT and plate agglutination. Confirmed cases are probable cases with one positive result among the following tests: SAT, complement fixation test, Coombs test, or isolation of *Brucella* spp*.* [1]. The sensitivity of the confirmatory agglutination tests for brucellosis was influenced by the cut-off value used and the local population’s infection rate. The sensitivity of SAT could be as high as 84.6% for acute brucellosis [4].

A case was classified as timely diagnosis if the patient received confirmation of human brucellosis within 30 days from the date of onset, while a case was classified as delayed diagnosis if the patient received confirmation of human brucellosis more than 30 days from the date of onset [37, 38]. Onset means that the patient presents the suspected symptoms such as fever, fatigue, nausea, vomiting, malaise, headache, myalgia, and arthralgia, or signs such as enlarged lymph nodes and swelling of testis.

### Descriptive analysis and mapping

We used cumulative incidence (CUI) to describe the disease frequency during the study period. CUI was calculated by the number of new cases during a period divided by the number of subjects at risk in the population at the beginning of the year. The temporal trend was described by yearly CUI. Graph on both the monthly number and monthly incidence was drawn to show the seasonality of human brucellosis. We explored the temporal trend and population pattern of human brucellosis using SAS 9.3 software (SAS Institute Inc., Cary, NC, USA). Disease maps of human brucellosis from 2005 to 2014 in Shanxi Province were visualized using ArcGIS 10.3 (ESRI, Redlands, CA, USA) based on prefecture, county and township boundary respectively. High-risk areas mean counties and towns with high CUI.

### Retrospective space-time scan statistical analysis

The space-time scan statistic (STSS) [39, 40] has been widely used to describe the spatial-temporal pattern of disease in both retrospective [40, 41] and prospective ways [42, 43]. The STSS utilizes thousands or millions of overlapping cylinders to define the scanning window, each being a possible candidate for an outbreak. The circular base represents the geographical area of the potential outbreak [39]. The height reflects the possible cluster’s time interval. The base and height of the cylinders (scanning windows) are in dynamic change to detect any possible space-time cluster. Monte Carlo simulations are operated to access the *p*-value using a Poisson model. The null hypothesis of this model is that there existed no difference between the incidence rate inside and outside the cylinders. Log Likelihood Ratio (LLR) and relative risk (RR) are calculated to test the hypothesis for each scanning window. The cluster with the maximum LLR is the primary cluster and the rest with smaller but statistically significant (*P* < 0.001 in this study) are the secondary clusters. In our study the denominator used in the Poisson model is the yearly population of each township not stratified by age group with the yearly case number of each township as numerator.

We used Satscan software 9.4 (http://www.satscan.org/) to conduct the retrospective STSS analysis of the space-time cluster in our study. This software is developed by Martin Kulldorff, Harvard Medical School (Boston, USA) and the Information Management Services Inc. (Maryland, USA). In our study, the spatial scope was 1398 towns in Shanxi Province and the temporal scope was from 2005 to 2014. We explored the space-time cluster for each year for understanding the spatial-temporal pattern of each year and the change during this decade. Therefore, the timeframe for each experiment was one year, which was also good to control the temporal trend. That meant we run ten scanning tests. In each test we used one year as a study period and aimed to detect the space-time clusters for that year. Based on the ten tests’ results we could explore the space-time clusters for each year and see how these clusters are changing over years both at temporal &geographical dimensions. As it is lack of criteria on the cluster size [43], we used 20% studied population as the maximum cluster size, setting time interval as of less than or equal to half the timeframe (default). The dataset used for Satscan can be found in the Table S7 of Additional file 2.

## Results

### Temporal trend and seasonality

**Fig. 1:**
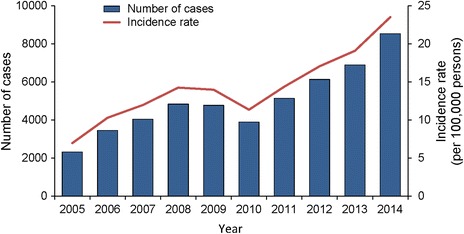
Annual reported cases and CUI of human brucellosis in Shanxi Province, 2005–2014

**Fig. 2:**
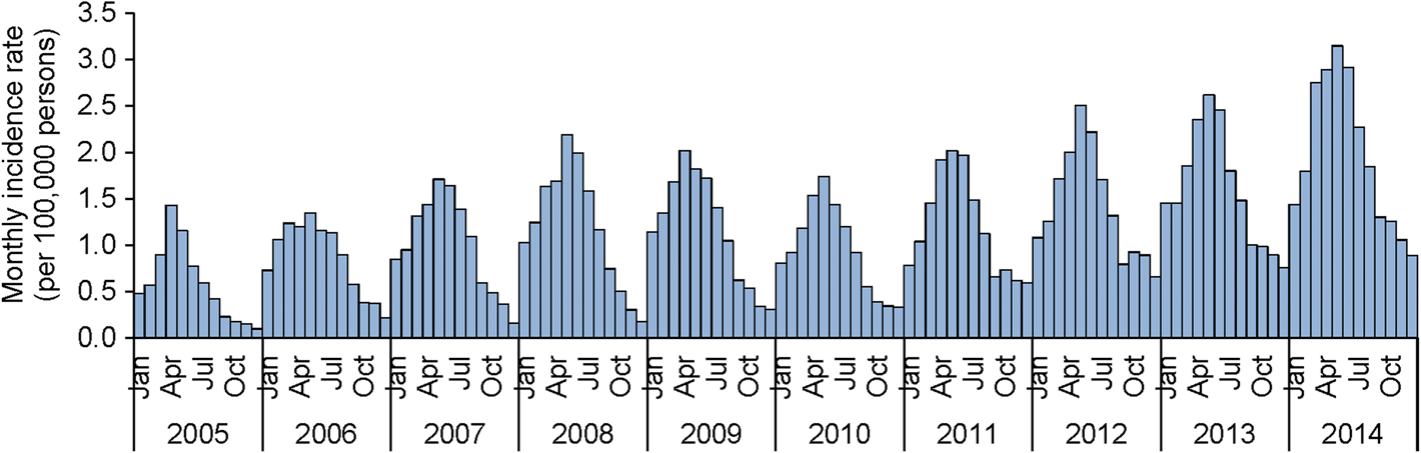
Monthly reported incidence rate of human brucellosis in Shanxi Province, 2005–2014

The lowest recorded CUI of human brucellosis in Shanxi Province during the past decade was 7.0/100,000 in 2005 with 2320 cases of the disease. Except for a slight decline in both the number of cases and CUI in 2009 and 2010, Shanxi’s epidemic trend of human brucellosis has sharply increased during the past decade, with an average annual increase in CUI of 14.5%. In 2014, the incidence rate of human brucellosis in the province reached 23.5/100,000 with 8540 reported cases, a highest record during 2005–2014 in Shanxi (Fig. 1). A seasonality of human brucellosis has been found in Shanxi Province, with high incidence in late spring, the whole summer and a seasonal peak in May (Fig. 2).

### Geographic distribution

**Fig. 3:**
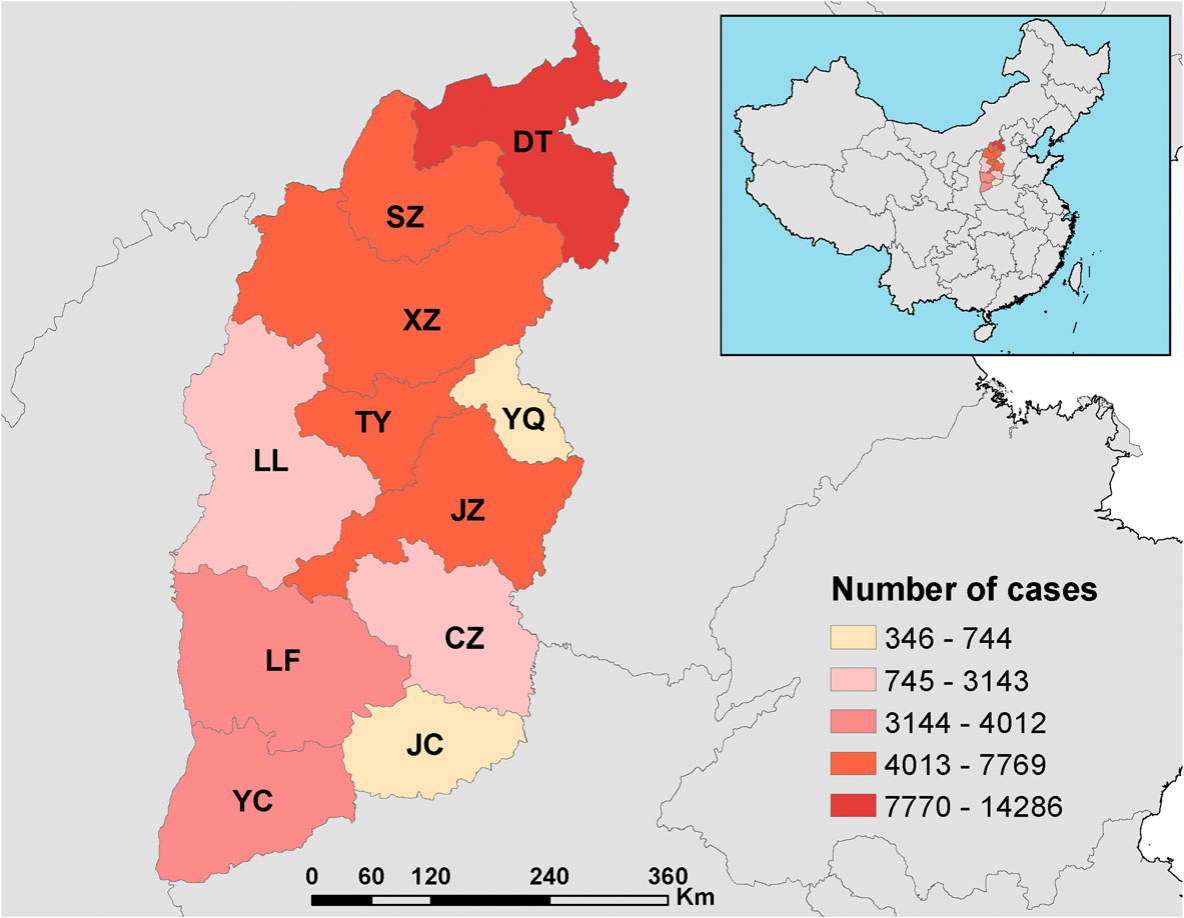
Cumulative distribution of human brucellosis cases at the prefectural level in Shanxi Province, 2005–2014. TY = TaiYuan City; DT = DaTong City; YQ = YangQuan City; CZ = ChangZhi City; SZ = ShuoZhou City; XZ = XinZhou City; LL = LvLiang City; JZ = JinZhong City; YC = YunCheng City; LF = LinFen City; JC = JinCheng City

A total of 50,002 cases of human brucellosis were reported in Shanxi Province from 2005 to 2014. The prefectures of Datong (with 14,286 cases) and Shuozhou (with 7769 cases) had the most accumulated cases among all 13 prefectures during that period, accounting for 44.1% of the total cases (50,002) in the province. These two prefectures are located in the northwestern Shanxi, adjacent to the Inner Mongolia Autonomous Area, another Chinese province with the highest number of brucellosis cases (Fig. 3, Table S1 in Additional file 2).

Human brucellosis cases were reported among 104 of 119 counties in Shanxi Province in 2005 and in all 119 counties in 2014. There were 439 (31.5%) towns with cases reported in 2005, while this number reached 1153 (82.5%) in 2014 (Table S1 of Additional file 2).

### Socio-demographic characteristics

During the study period, the median age of all human brucellosis patients in Shanxi Province was 49.0 years (IQR: 39.0–58.0). Although all population regardless of age is equally susceptible to the disease, the majority of patients was aged from 25 to 64 years with a proportion as high as 81.9%. Children (≤14 year-old) accounted for 2.3% of cases and the elderly (≥65 year-old) for 10.8% (Table S2 of Additional file 2). There were much more male (*n* = 39,798) patients than female patients (*n* = 10,204) in Shanxi Province, with a sex ratio of 3.9:1 (Table S2 of Additional file 2).

### Health service-seeking behavior

**Fig. 4:**
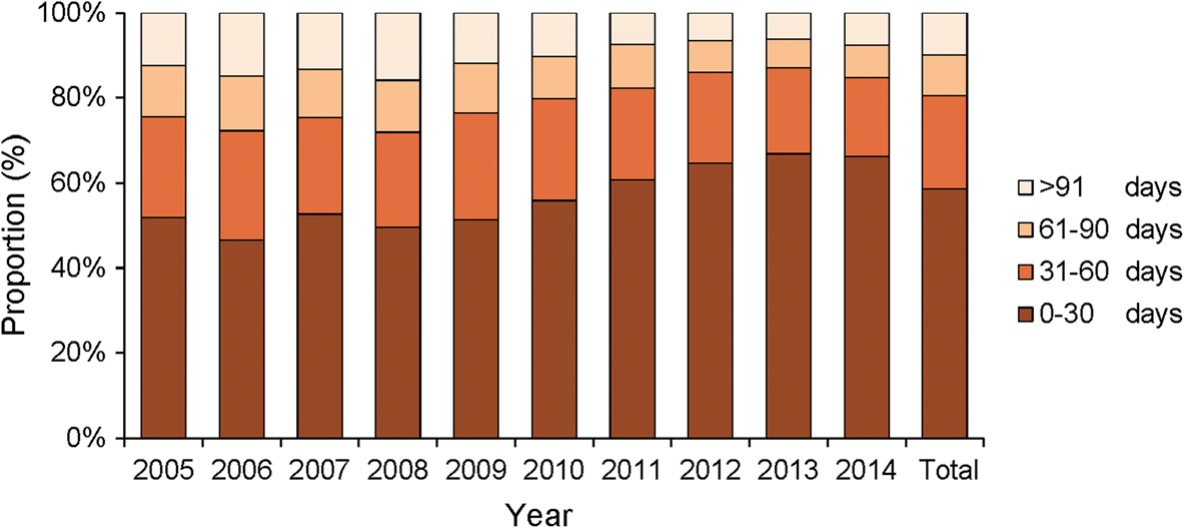
Temporal distribution from onset date to diagnosis date, of reported cases in Shanxi Province, 2005 –2014; more than 30 days was considered delayed diagnosis

From 2005 to 2014, 41.4% of human brucellosis patients obtained diagnosis beyond 30 days from the date of onset. Even 19.5% of the patients received a diagnosis more than 60 days from illness onset. Although the proportion of patients with delayed diagnosis decreased during the study period, 33.8% of patients still delayed their confirmation of human brucellosis in 2014, and 15.3% of cases even delayed more than 2 months from onset (Fig. 4, Table S2 of Additional file 2).

### High-risk area change based on county and town polygons

**Fig. 5:**
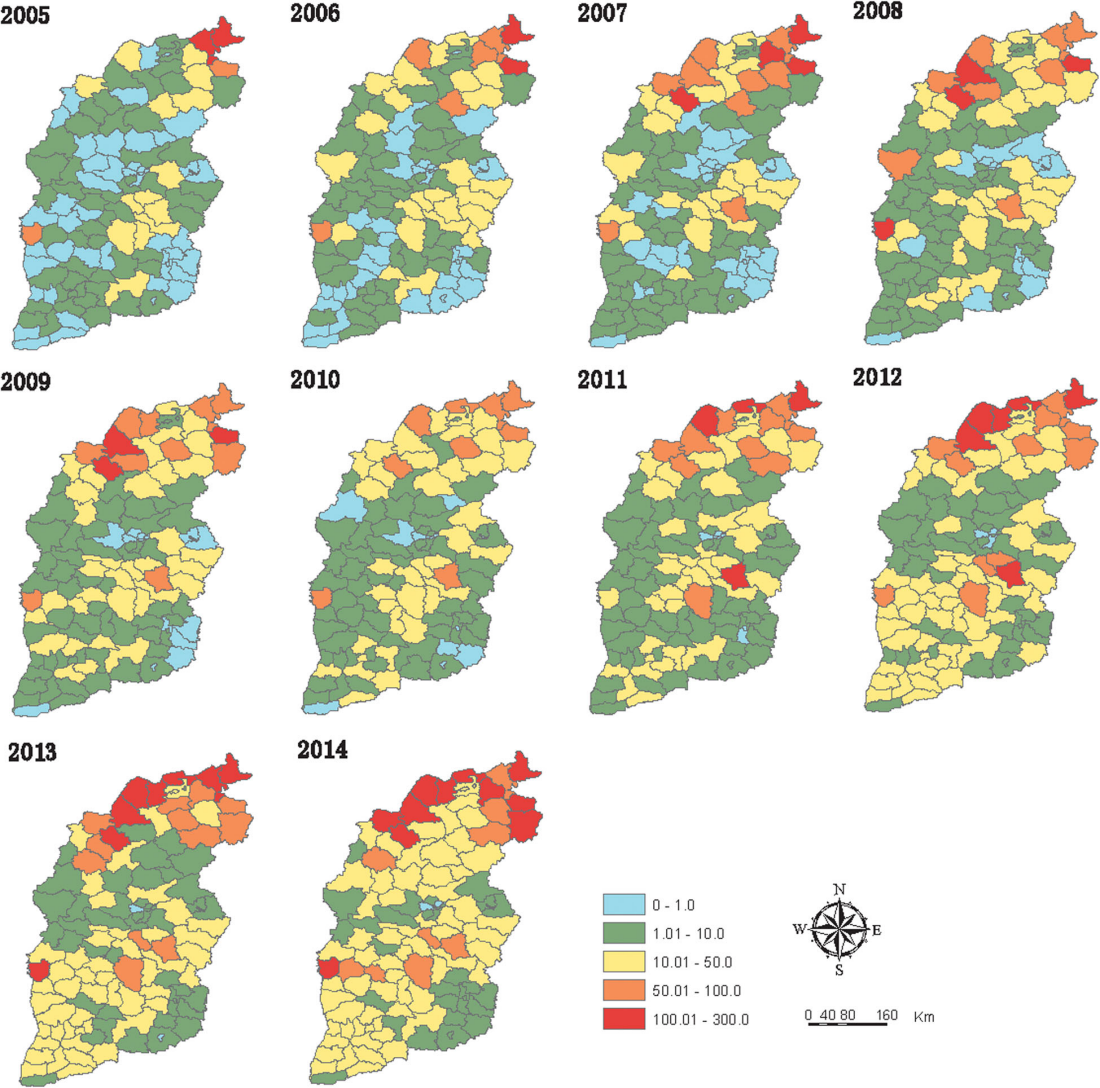
CUI distribution of human brucellosis in Shanxi Province, based on county polygons (2005–2014)

During the past decade both the number of yearly reported case and CUI varied among counties. In 2005, the median of number of case and CUI were 4 cases (IQR: 1–19) and 1.4/100,000 (IQR: 0.3/100,000–1.4/100,000) respectively, which increased to 42 cases (IQR: 18–115) and 14.9/100,000 (IQR: 5.3/100,000–31.8/100,000) in 2014. In 2005, counties located in the north of Shanxi Province had the highest CUI (over 100.0/100,000) with a proportion of 1.7%, and 15.5% counties had CUI over 10.0/100,000. In 2009, those counties with the highest CUI shifted to northwest and slightly southward, whereas the percentage of counties with CUI greater than 10.00/100,000 increased to 39.5%. In 2014, the proportion of counties with the highest CUI (over 100.0/100,000) increased to 9.2% and were still located in the north of the province, while 70.6% of counties’ CUI exceeded 10.0/100,000 (Fig. 5, Table S3 and Figure S3 of Additional file 2).

**Fig. 6:**
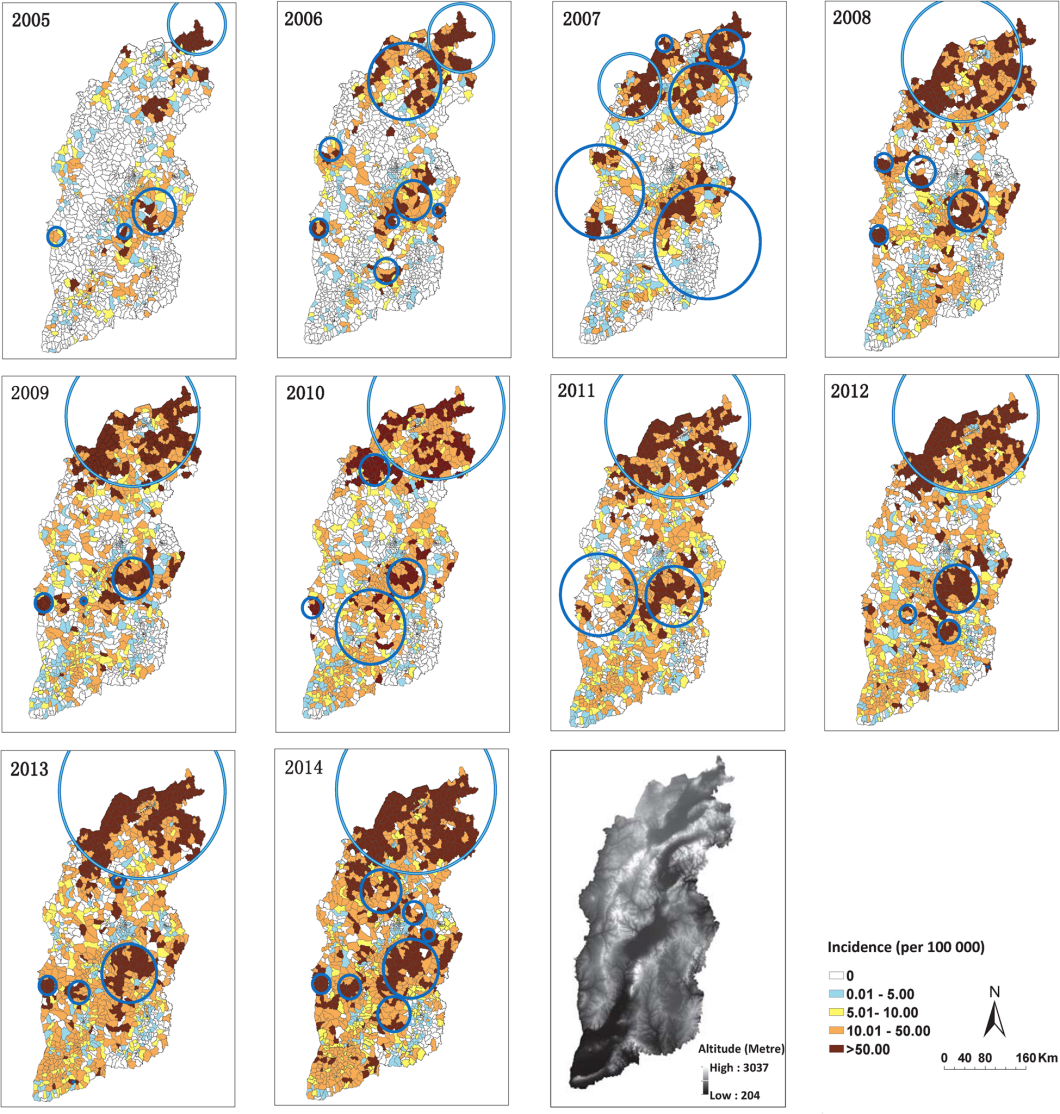
Annual distribution and clusters of reported cases of human brucellosis in Shanxi Province, based on town polygons (2005–2014). ○ means cluster. The clusters with *light blue color* were the primary clusters, which meant the most likely clusters. There was one primary cluster for each year with the largest LLR, while others were secondary clusters

At township level in 2005, there were 212 (15.3%) towns with CUI over 10.0/100,000, gathering in the northern part of Shanxi; while 72 (5.2%) towns with CUI over 50.0/100,000 scattered throughout the province. In 2009, there were 511 (36.6%) towns with CUI over 10.0/100,000 and 174 (12.5%) towns with CUI over 50.0/100,000 respectively, most of which have extended to more areas in the north; others were concentrated in the center of the province or had shifted slightly southward, looking like aggregating in a diagonal line across the center and south of Shanxi. In 2014, there were 887 (59.9%) towns with CUI over 10.0/100,000, involving most of the areas of the whole province, while 337 (24.1%) towns with CUI over 50.0/100,000 involved the majority of northern areas and also extended to more areas in the center (Fig. 6, Table S4 and Figure S4 of Additional file 2).

During last decade, high-risk areas were initially found among several towns located in the north of the province; and then expanded to more towns in the north. High-risk areas spread to the center and then to the south of Shanxi, concentrating in agricultural basins and the adjacent highlands and affecting more towns (Fig. 6).

### Space-time clusters based on town polygons

There were 55 space-time clusters of human brucellosis detected during the past decade. It seems that clusters of human brucellosis can happen in any month and any season during a year (Table S8 of Additional file 2). The clusters located in the high risk areas. They moved from north, towards middle and to the south in the past decade, the location and time period of which might change during different years. However, some clusters’ location is relatively steady despite of time changing (Fig. 6, Table S8 of Additional file 2).

## Discussion

In this study, NIDRIS data were extracted to describe the epidemic characteristics of human brucellosis in Shanxi Province. High-risk areas were explored at the township level to elucidate the necessity for decentralization of disease control activities to lower administrative levels, such as the township level, particularly in high-risk areas.

Based on the result of this study, the epidemic of human brucellosis in Shanxi Province has been on the rise over the past decade with a rapidly increasing incidence and expansion of affected areas. This trend is consistent with the profile of the whole nation [21, 22, 24, 31] and other northern provinces, such as the Inner Mongolia Autonomous Area [25] and Shandong Province [27]. Sero-prevalence studies have also indicated an increasing drift in both livestock (0.7% in 2005–2006, and 1.5% in sheep in 2009, 3.71% in 2014) and occupational population (13.3% in 2005–2006, 22.0% in 2007, and 22.8% in 2011) [18–20, 44]. This trend may be attributed to the speedy development of husbandry in the recent years, especially goat and sheep breeding in northern rural China [26, 32]. More than 90% of human brucellosis cases in the country are infected by contacting the ill sheep and goats [45, 46]. Rural populations, especially those living in northern China, made their living by raising sheep, goats and cattle. It is common for rural farmers to share a living space, such as a yard, with their livestock. Unfortunately they seldom wear personal protective equipment such as gloves, aprons, or masks [47, 48]. These breeding way and behavior habit can easily cause exposure and infection when they directly contact the infected livestock. However, more research is needed to determine the driving factors behind the rising trend of human brucellosis.

The epidemic season in Shanxi Province is late spring and summer, consistent with that of the entire country [18–20, 24, 26]. This time period coincides with the lambing season, which may cause greater opportunities of exposure to *Brucella*. Most brucellosis patients are young and middle-aged men, who are the primary money earners in rural families and the backbone of the labor force. However, almost half of patients had delayed diagnosis, thereby missing the optimal treatment windows [49] and increasing the risk of developing chronic brucellosis. Chronic disease would cause greater economic loss and health harm [50–52] for affected individuals and their families, even the whole society. Launching awareness campaigns before the epidemic season may be effective to prevent the risk group from infection and promote them to seek timely treatment when infected. More research is required to clarify the disease burden of human brucellosis in China, so as to mobilize greater resources to combat this disease. One health approach is optimal and necessary to contain this neglected zoonosis, which need the close coordination from health and agriculture sectors. It is worth mentioning that stopping animal disease is the principal strategy for human brucellosis control. The Quarantine-Slaughter-Immunization strategy had been proven effective last century, which is still performed but needed inputting much more resource and effort in China [46]. In 2006 the immunization rate in sheep was 59.9% (67.7 million/113.0 million) and the quarantine rate was 4.8% (135,841/2,830,013) in Shanxi Province [53]. In addition, we found a much higher proportion of male patients in Shanxi Province than that in entire country [26], which might be attributed to the different exposure risk.

The disease maps analyzed at the township level during the study period had indicated that the high-risk areas of human brucellosis concentrated in certain towns of Shanxi Province and had spread from north to south, aggregating in agricultural basins and surrounding areas with low to middle elevation. This implies that brucellosis may be imported from the Inner Mongolia Autonomous Area, which shares Shanxi’s northern border. Another study in China revealed that human brucellosis was most prevalent in grassland areas with elevations between 800 and 1600 m [26]. More research should be conducted to determine those factors impacting the spatial distribution of this disease.

Our finding that nearly one thirds of the cases had delayed diagnosis in 2014 suggests the necessity of decentralizing basic disease prevention and control units to the town or even village level, in order to increase accessibility to the diagnosis and treatment service. Currently, the public health system in rural China consists of three levels: county-level CDCs and hospitals, township hospitals, and village clinics. In most areas of China, the basic control and prevention units of human brucellosis are county-level CDCs and hospitals; however, many patients are farmers living in the countryside [18–20], where located very far from these CDCs and hospitals. Measures must be taken to accelerate the accessibility of diagnosis and treatment service of human brucellosis in these remote rural areas.

A cluster means a potential outbreak. There existed a few potential outbreaks of human brucellosis in the past decade according to the results of this study. Therefore, we should consider the essentiality of including human brucellosis in the disease list of the National Infectious Disease Alerting and Warning System in China. Although human brucellosis represents seasonality in terms of incidence, it seems that the clusters could occur at any season. This indicates that either the transmission channel or the impacting population of the clusters may be various. We should pay more attention to the potential outbreaks that may be caused by contaminated milk and the related products, which may involve all population including the elder, the children and women rather than confining to the occupational groups, who are commonly infected by contact with ill animals [1, 2].

There are several limitations in this study. Firstly, the data used were collected from passive public health surveillance. The data quality may be influenced by the key steps in surveillance including reporting methods, availability of health facilities and laboratory diagnostics, underreporting, completeness and accuracy of data over the years. Especially, human brucellosis’s signs and symptoms are normally untypical, which might lead to misdiagnose and underreport for this disease. Secondly, access to health service and the capacity of the health provider maybe varied by time, which may partly impact the temporal trend of the incidence. Lastly, some codes for towns in the case data set could not be matched to those in the map data set; therefore, those towns failed to appear on the disease maps. However, there were not many unmatched towns, less than one-tenth of the total, so the representativeness was not discounted.

## Conclusion

From 2005 to 2014, the incidence of human brucellosis in Shanxi Province of China has risen and expanded to more areas. High-risk areas were initially limited at some towns in the north of the province, and then gradually expended to the center and south of Shanxi, particularly in agricultural and surrounding areas. Quite a few human brucellosis cases still failed to obtain timely diagnosis. More resources should be devoted to enhancing control efforts in high-risk areas, and decentralized to the township level to facilitate prompt treatment in epidemic regions, such as Shanxi Province. In addition, more researches are needed to identify the drivers behind the increasing trend of this disease.

## Additional files

Additional file 1: Summary of diagnostic criteria for brucellosis in mainland China since 1995. (DOCX 35 kb)
Additional file 2: Supplementary tables and figures. (DOCX 74 kb)

## Abbreviations

CDC: Center for disease control and prevention
CUI: Cumulative incidence
GIS: Geographic information System
NIDRIS: Notifiable infectious disease reporting information system
STSS: Space-time scan statistic
