# Supplementary material for: Epidemic characteristics, high-risk townships and space-time clusters of human brucellosis in Shanxi Province of China, 2005–2014
Source: BMC Infect Dis. 2016 Dec 19;16:760. doi: 10.1186/s12879-016-2086-x (PMC5165709; doi:10.1186/s12879-016-2086-x)
Supplement: Additional file 1: — Summary of diagnostic criteria for brucellosis in mainland China since 1995. (DOCX 35 kb) [file 12879_2016_2086_MOESM1_ESM.docx]

**Additional file1: Summary of Diagnostic Criteria for Brucellosis in mainland China since 1995**

| \| **Epidemiologic linkage** \| \| --- \| | 1.1 Before the onset of illness, the case had a history of close contact with suspected/confirmed animal cases, contaminated animal products, or cultures of Brucella spp.; or living in endemic areas; or closely connected with the produce, usage and research of vaccine of Brucella spp. (since 1995) | |
| --- | --- | --- | --- |
| **Clinical description** | 2.1 Present with undulant, continued, intermittent or irregular fever (including low fever) of variable duration (some days or weeks), with profuse sweating, fatigue, muscle pain, arthralgia, etc. lympademia, splenomegaly and hepatomegaly, orchitis and epididymitis are common, but small number of patients may present with jaundice and a variety of rashes; The osteoarticular complications are common in patients at chronic phase. (since 1995) | |
| **Laboratory tests** | 3.1 Presumptive diagnosis   - Positive results of plate agglutination test (PAT) or Bengal plate agglutination test (RBPT): 0.03ml serum (++) (since 1996). - Intradermal allergic reaction test by 0.1ml brucellin: skin redness and infiltration range ≥ 2.0cm×2.0cm or 4.0cm^2^ observed at 24h or 48h (since 1996).   3.2 Serological diagnosis   - Standard tube agglutination test (SAT): 1:100++ or higher in titer (since 1996); or 1:50++ or higher in titer for the patient with course over one year (since October 2007); or ≥4-fold rise in titer in the paired sera after 2-4 weeks of the first test 1:100++ or higher in titer of the patients with a history of Brucella vaccination within half year (since 1996). - Complement fixation test (CFT): 1:10++ or higher in titer (since 1996). - Coomb’s test: 1:400++ or higher in titer (since 1996).   3.3 Isolation of Brucella spp. from blood or other clinical specimen (since 1996). | |
| **Diagnostic Criteria and Principles of Management for Human Brucellosis (GB 15988-1995)** | | Suspected cases: 1.1+2.1  Clinic/probable cases: 1.1+2.1+3.1  Confirmed cases: 1.1+2.1+3.1+any of (3.2,3.3)  Latent infection: None |
| **Diagnostic Criteria for Brucellosis (WS 269-2007)** | | Suspected cases: 1.1+2.1  Clinic/probable cases: 1.1+2.1+any of 3.1  Confirmed cases: 1.1+2.1+3.1+any of (3.2,3.3)  Latent infection: 1.1+any of (3.2, 3.3) without 2.1 |
| **Guidelines for Diagnosis and Treatment of Human Brucellosis (2012)** | | Suspected cases: 1.1+2.1  Clinic/probable cases: 1.1+2.1+ any of 3.1  Confirmed cases: 1.1+2.1+3.1+any of (3.2,3.3)  Latent infection: 1.1+any of (3.2, 3.3) without 2.1 |

*Diagnostic Criteria and Principles of Management for Human Brucellosis (GB 15988-1995) & Diagnostic Criteria for Brucellosis (WS 269-2007)

**Base on previous criteria, Guidelines for Diagnosis and Treatment of Human Brucellosis (2012) issued by MOH of China in 2012 improve the diagnosis and case classification and laboratory tests.
