# Supplementary material for: Epidemic characteristics, high-risk townships and space-time clusters of human brucellosis in Shanxi Province of China, 2005–2014
Source: BMC Infect Dis. 2016 Dec 19;16:760. doi: 10.1186/s12879-016-2086-x (PMC5165709; doi:10.1186/s12879-016-2086-x)
Supplement: Additional file 2: — Supplementary tables and figures. (DOCX 74 kb) [file 12879_2016_2086_MOESM2_ESM.docx]

## Additional file 2: supplementary tables and figures

**Figure 1. Number and percentage of towns with reported cases in Shanxi Province, China, 2005-2014**

**Table 1.** **Number and percentage of counties and towns with reported cases in Shanxi Province, China, 2005-2014**

| year | counties with reported cases | towns with reported cases |
| --- | --- | --- |
| 2005 | 104 (87.4%) | 478 (34.4%) |
| 2006 | 107 (89.9%) | 604 (43.3%) |
| 2007 | 110 (92.4%) | 689 (49.3%) |
| 2008 | 110 (92.4%) | 823 (58.9%) |
| 2009 | 115 (96.6%) | 869 (62.2%) |
| 2010 | 117 (98.3%) | 876 (62.7%) |
| 2011 | 119 (100.0%) | 974 (69.7%) |
| 2012 | 118 (99.2%) | 1046 (74.9%) |
| 2013 | 119 (100.0%) | 1104 (79.0%) |
| 2014 | 119 (100.0%) | 1206 (86.3%) |

**Figure 2. Age and gender distribution among accumulative cases in Shanxi Province, 2005-2014**

**Table 2.** **Characteristics of human brucellosis cases in Shanxi Province, China (2005–2014)**

| Characteristic | Total (n=50 002) |  | Characteristic | Total (n=50 002) |
| --- | --- | --- | --- | --- |
| Type of case | |  | Month of onset | |
| Confirmed | 39 203 (78.60%)* |  | February | 4063(8.13) |
| Probable | 10 799 (21.40%) |  | March | 5491(10.98) |
| Gender | |  | April | 6453(12.91) |
| Male | 39 798 (79.60%) |  | May | 7075(14.15) |
| Female | 10 204 (20.40%) |  | June | 6399(12.80) |
| Age | |  | July | 5091(10.18) |
| Median (years, IQR) | 49.03 (39.03–58.04) |  | August | 3957(7.91) |
| Age group |  |  | September | 2474(4.95) |
| 0-14 | 1153 (2.31%) |  | October | 2237(4.47) |
| 15-24 | 2490 (4.98%) |  | November | 1876(3.75) |
| 25-34 | 4979 (9.96%) |  | December | 1474(2.95) |
| 35-44 | 10 492 (20.98%) |  | Median of diagnosis delay (from onset to diagnosis) | |
| 45-54 | 13 590 (27.18%) |  | Median (IQR) | 23(10-46) |
| 55-64 | 11 880 (23.76%) |  | 0-30days | 29282(58.65) |
| 65 and above | 5418 (10.83%) |  | 31-60days | 10896(21.82) |
| Month of onset | |  | 61-90days | 4814(9.64) |
| January | 3412(6.82) |  | >91days | 4933(9.88) |

Abbreviation: IQR, inter-quartile range. Data are presented as no. (%) of patients unless otherwise indicated. ^*^ Data are presented as no. (%) of patients unless otherwise indicated.

**Table 3. The categories of counties based on CUI in Shanxi Province,2005-2014**

(Unit:/100,000)

| year | 0.00-1.00 | 1.01-10.00 | 10.01-50.00 | 50.01-100.00 | 100.01-500.00 |
| --- | --- | --- | --- | --- | --- |
| 2005 | 48(40.3%) | 53(44.5%) | 14(11.8%) | 2(1.7%) | 2(1.7%) |
| 2006 | 37(31.1%) | 51(42.7%) | 24(20.2%) | 5(4.2%) | 2(1.7%) |
| 2007 | 33(27.7%) | 51(42.7%) | 22(18.5%) | 9(7.6%) | 4(3.4%) |
| 2008 | 18(15.1%) | 59(49.6%) | 30(25.2%) | 8(6.7%) | 4(3.4%) |
| 2009 | 15(12.6%) | 57(47.9%) | 34(28.6%) | 10(8.4%) | 3(2.5%) |
| 2010 | 12(10.2%) | 61(51.7%) | 36(30.5%) | 9(7.6%) | 0(0.0%) |
| 2011 | 6(5.0%) | 63(52.9%) | 36(30.3%) | 10(8.4%) | 4(3.4%) |
| 2012 | 5(4.2%) | 46(39.0%) | 50(42.4%) | 11(9.3%) | 6(5.1%) |
| 2013 | 2(1.7%) | 47(39.5%) | 50(42.0%) | 12(10.1%) | 8(6.7%) |
| 2014 | 3(2.5%) | 32(26.9%) | 64(53.8%) | 9(7.6%) | 11(9.2%) |

**Figure 3.** **The categories of counties based on CUI in Shanxi Province, 2005-2014**

**Table 4. The categories of towns based on CUI in Shanxi Province,2005-2014**

(Unit: per 100000)

|  | 0 | 0.01～5.00 | 5.01～10.00 | 10.01～50.00 | >50.00 |
| --- | --- | --- | --- | --- | --- |
|  | N(%) | N(%) | N(%) | N(%) | N(%) |
| 2005 | 952 (68.4%) | 116 (8.3%) | 111 (8.0%) | 140 (10.1%) | 72 (5.2%) |
| 2006 | 854 (61.4 %) | 110 (7.9%) | 93 (6.7%) | 218 (15.7%) | 119 (8.6%) |
| 2007 | 778 (55.7%) | 134 (9.6%) | 103 (7.4%) | 222 (15.9%) | 160 (11.5%) |
| 2008 | 616 (44.1 %) | 144 (10.3%) | 128 (9.2%) | 315 (22.5%) | 194 (13.9%) |
| 2009 | 581 (41.6%) | 156 (11.2%) | 149 (10.7%) | 337 (24.1%) | 174 (12.5 %) |
| 2010 | 559 (40.0%) | 144 (10.3%) | 158 (11.3%) | 391 (28.0 %) | 145 (10.4%) |
| 2011 | 461 (33.0%) | 167 (12.0 %) | 168 (12.0%) | 417 (29.8 %) | 184 (13.2%) |
| 2012 | 396 (28.3%) | 169 (12.1%) | 157 (11.2%) | 447 (32.0%) | 228 (16.3%) |
| 2013 | 326 (23.3%) | 166 (11.9%) | 163(11.7%) | 499(35.7%) | 244 (17.5%) |
| 2014 | 245 (17.5%) | 151 (10.8%) | 165 (11.8%) | 500 (35.8%) | 337 (24.1%) |

**Figure 4. The categories of towns based on CUI in Shanxi province, 2005-2014.**

**Table 5. Yearly gender ratio of reported cases Shanxi Province,2005-2014**

| Year | 2005 | 2006 | 2007 | 2008 | 2009 | 2010 | 2011 | 2012 | 2013 | 2014 |
| --- | --- | --- | --- | --- | --- | --- | --- | --- | --- | --- |
| N. of Male | 1872 | 2738 | 3247 | 3870 | 3780 | 3095 | 4103 | 4863 | 5379 | 6778 |
| N. of female | 439 | 684 | 785 | 946 | 957 | 790 | 992 | 1215 | 1516 | 1876 |
| gender ratio | 4.3 | 4.0 | 4.1 | 4.1 | 3.9 | 3.9 | 4.1 | 4.0 | 3.5 | 3.6 |

*N.: number of cases

| **Variables** | **Definition/classification** | **Completeness** |
| --- | --- | --- |
| ID | A unique 8-digital number for each case. | 100% reported |
| Gender | Male and Female | 100% reported |
| Age | The interval time from the date of birth to the date of onset | 100% reported |
| The zone code of address | A unique 6-digital number at county level | 100% reported |
| Nationality | Chinese or foreigner | 100% reported |
| Occupation | Occupation/status of cases | 99.5% reported |
| Type of diagnosis | Probable case (clinical diagnosed case)  Confirmed case (laboratory confirmed case) | 100% reported |
| Date of onset | The date of illness onset | 100% reported |
| Date of diagnosis | The date of diagnosis as a probable or confirmed dengue case | 100% reported |
| Date of report | The first date of reporting to dengue surveillance system | 100% reported |
| Date of Death | The date of case death, if applicable. | 100% reported |

**Table 6. The list of variables in the individual dataset of human brucellosis cases from 2005 to 2014.**

Note: The foreigners are not included in all analysis of this study.

**Table 7. T****he list of the variables of the dataset used for Satscan.**

| **File** | **Variables** | **Definition/classification** | **Completeness** |
| --- | --- | --- | --- |
| Case file | The zone code of address | A unique 6-digital number at county level | 100% |
|  | Date of onset | The date of illness onset | 100% |
|  | Number of case | We use the individual as the case file by only reserve the above two variables, add this variable, putting the number of “1” in this column, as one record means one case. | 100% |
| Population  file | The zone code of address | A unique 6-digital number at county level | 100% |
|  | date | The date of the population is identical for each year. | 100% |
|  | population | The population for each town was required from National Bureau of Statistics of the People's Republic of China. | 100% |
| Coordinates  file | The zone code of address | A unique 6-digital number at county level | 100% |
|  | latitude/Lat | The latitude of the centroid of the town/community the patient lived.  We used ArcGIS to generate “Lat” and “Long” based on the map information. | 100% |
|  | longitude/Long | The longitude of the centroid of the town/community the patient lived. | 100% |

**Table 8. Secondary space-time clusters of human brucellosis in Shanxi Province, 2005-2014.**

| Scan | Cluster Time | Centroid(latitude,longitude) | cluster towns | LLR | RR | *P*-Value |
| --- | --- | --- | --- | --- | --- | --- |
| Time frame |  | /Radius(km) |  |  |  |  |
| 2005 | Feb27-Jul 16 | (36.85N,112.32E)/14.77 | 5 | 67.51 | 14.43 | <0.001 |
| 2005 | Mar 27-Jul 11 | (36.76N,110.63E)/19.22 | 7 | 49.67 | 9.42 | <0.001 |
| 2005 | Mar 13-Sep 10 | (37.22N,113.06E)/46.79 | 27 | 28.58 | 2.49 | <0.001 |
| 2006 | Jan 1-Jun 18 | (39.46N,112.76E)/77.79 | 88 | 93.99 | 2.22 | <0.001 |
| 2006 | Jan 30-Jul 30 | (36.89N,112.45E)/13.65 | 4 | 60.80 | 18.37 | <0.001 |
| 2006 | Feb 27-Aug 27 | (37.09N,113.60E)/12.12 | 2 | 43.99 | 10.78 | <0.001 |
| 2006 | Feb 13-Jul 30 | (36.76N,110.63E)/19.22 | 7 | 43.83 | 6.15 | <0.001 |
| 2006 | Feb 27-Aug 27 | (37.28N,112.96E)/40.20 | 27 | 30.25 | 2.29 | <0.001 |
| 2006 | Apr 10-Sep 24 | (38.22N,110.91E)/23.19 | 7 | 27.35 | 3.56 | <0.001 |
| 2006 | Jan 16-Jun 4 | (35.96N,112.29E)/26.13 | 6 | 18.53 | 5.79 | <0.001 |
| 2007 | Jan 1-Jun 4 | (40.05N,113.88E)/38.66 | 29 | 147.75 | 3.31 | <0.001 |
| 2007 | Mar 27-Jul 2 | (36.50N,113.43E)/117.45 | 74 | 83.70 | 3.17 | <0.001 |
| 2007 | Jan 16-Jul 16 | (39.15N,113.32E)/71.95 | 77 | 80.85 | 1.92 | <0.001 |
| 2007 | May 8-Aug 13 | (37.44N,110.76E)/95.58 | 60 | 48.79 | 2.43 | <0.001 |
| 2007 | Jan 16-Jun 18 | (40.15N,112.36E)/16.63 | 3 | 26.31 | 5.66 | <0.001 |
| 2008 | Jan 1-Jul 2 | (38.09N,110.75E)/18.78 | 5 | 198.84 | 12.45 | <0.001 |
| 2008 | Feb 14-Aug 13 | (37.21N,112.83E)/42.15 | 34 | 115.34 | 3.23 | <0.001 |
| 2008 | Feb 14-Aug 13 | (36.76N,110.63E)/19.22 | 7 | 79.65 | 8.42 | <0.001 |
| 2008 | Oct 23-Nov 5 | (37.92N,111.66E)/31.78 | 7 | 20.53 | 20.16 | <0.001 |
| 2009 | Jan 16-Jul 16 | (37.21N,112.83E)/42.57 | 35 | 170.48 | 3.98 | <0.001 |
| 2009 | Mar 27-May 21 | (36.76N,110.63E)/19.22 | 7 | 38.27 | 11.55 | <0.001 |
| 2009 | Apr 10-Aug 27 | (36.80N,111.62E)/6.43 | 2 | 30.70 | 15.54 | <0.001 |
| 2010 | Jan 30-Jul 30 | (37.21N,112.83E)/40.08 | 32 | 186.46 | 4.73 | <0.001 |
| 2010 | Jan 1-Jul 2 | (39.21N,112.05E)/31.53 | 20 | 178.24 | 9.38 | <0.001 |
| 2010 | Mar 27-Jun 18 | (36.67N,110.49E)/21.05 | 6 | 42.46 | 11.38 | <0.001 |
| 2010 | Apr 10-Jun 18 | (36.31N,111.95E)/77.02 | 110 | 20.22 | 1.77 | <0.001 |
| 2011 | Feb 27-Aug 13 | (37.04N,112.85E)/62.19 | 71 | 332.92 | 4.30 | <0.001 |
| 2011 | Apr 10-Jun 18 | (37.07N,110.98E)/84.24 | 85 | 21.94 | 1.89 | <0.001 |
| 2012 | Feb 28-Jul 30 | (37.14N,112.75E)/49.21 | 56 | 365.80 | 4.82 | <0.001 |
| 2012 | Jan 31-Jul 16 | (36.34N,112.56E)/23.83 | 10 | 43.93 | 4.95 | <0.001 |
| 2012 | Feb 14-Jul 30 | (36.68N,111.53E)/17.72 | 7 | 39.95 | 5.53 | <0.001 |
| 2012 | Jul 3-Nov 19 | (36.78N,110.50E)/0.00 | 1 | 34.12 | 30.11 | <0.001 |
| 2012 | Mar 27-Aug 27 | (35.69N,113.52E)/0.00 | 1 | 19.71 | 15.29 | <0.001 |
| 2013 | Feb 27-Jul 16 | (36.99N,112.65E)/60.71 | 87 | 263.09 | 3.35 | <0.001 |
| 2013 | Feb 13-Jul 30 | (36.76N,110.63E)/19.22 | 7 | 91.46 | 9.42 | <0.001 |
| 2013 | Feb 27-Aug 27 | (34.97N,111.27E)/0.00 | 1 | 32.53 | 10.15 | <0.001 |
| 2013 | Feb 13-Jul 2 | (36.63N,111.40E)/23.28 | 14 | 29.11 | 3.47 | <0.001 |
| 2013 | Jul 31-Dec 17 | (38.68N,112.37E)/12.70 | 3 | 24.46 | 11.50 | <0.001 |
| 2014 | Feb 27-Jul 30 | (37.04N,112.85E)/61.12 | 86 | 183.99 | 2.73 | <0.001 |
| 2014 | Mar 13-Sep 10 | (36.76N,110.63E)/19.76 | 8 | 154.35 | 10.21 | <0.001 |
| 2014 | Feb 27-Aug 27 | (36.70N,111.33E)/24.75 | 14 | 93.81 | 4.86 | <0.001 |
| 2014 | Feb 27-Jul 16 | (38.47N,112.11E)/43.80 | 36 | 55.89 | 3.10 | <0.001 |
| 2014 | Feb 27-June 4 | (36.20N,112.43E)/36.38 | 20 | 23.78 | 2.88 | <0.001 |
| 2014 | Mar 27-May 7 | (37.66N,113.29E)/12.88 | 3 | 23.04 | 17.52 | <0.001 |
| 2014 | Feb 27-Aug 27 | (38.07N,112.93E)/23.21 | 10 | 21.74 | 2.88 | <0.001 |
